# Targeted apoptosis in ovarian cancer cells through mitochondrial dysfunction in response to *Sambucus nigra* agglutinin

**DOI:** 10.1038/cddis.2017.77

**Published:** 2017-05-04

**Authors:** Shreya Roy Chowdhury, Upasana Ray, Bishnu P Chatterjee, Sib S Roy

**Affiliations:** 1 Cell Biology and Physiology Division, CSIR-Indian Institute of Chemical Biology, Council of Scientific and Industrial Research, Kolkata, India; 2 Department of Natural Sciences, West Bengal University of Technology, Kolkata, India

## Abstract

Ovarian carcinoma (OC) patients encounter the severe challenge of clinical management owing to lack of screening measures, chemoresistance and finally dearth of non-toxic therapeutics. Cancer cells deploy various defense strategies to sustain the tumor microenvironment, among which deregulated apoptosis remains a versatile promoter of cancer progression. Although recent research has focused on identifying agents capable of inducing apoptosis in cancer cells, yet molecules efficiently breaching their survival advantage are yet to be classified. Here we identify lectin, *Sambucus nigra* agglutinin (SNA) to exhibit selectivity towards identifying OC by virtue of its specific recognition of *α*-2, 6-linked sialic acids. Superficial binding of SNA to the OC cells confirm the hyper-sialylated status of the disease. Further, SNA activates the signaling pathways of AKT and ERK1/2, which eventually promotes de-phosphorylation of dynamin-related protein-1 (Drp-1). Upon its translocation to the mitochondrial fission loci Drp-1 mediates the central role of switch in the mitochondrial phenotype to attain fragmented morphology. We confirmed mitochondrial outer membrane permeabilization resulting in ROS generation and cytochrome-c release into the cytosol. SNA response resulted in an allied shift of the bioenergetics profile from Warburg phenotype to elevated mitochondrial oxidative phosphorylation, altogether highlighting the involvement of mitochondrial dysfunction in restraining cancer progression. Inability to replenish the SNA-induced energy crunch of the proliferating cancer cells on the event of perturbed respiratory outcome resulted in cell cycle arrest before G2/M phase. Our findings position SNA at a crucial juncture where it proves to be a promising candidate for impeding progression of OC. Altogether we unveil the novel aspect of identifying natural molecules harboring the inherent capability of targeting mitochondrial structural dynamics, to hold the future for developing non-toxic therapeutics for treating OC.

The ever-climbing incidence rate of ovarian carcinoma (OC) makes it the most lethal female reproductive disorder. The disease is crippled by severe hurdles including lack of specific symptoms, no screening procedures and chemoresistance.^1, 2^ Majority of the cases are detected at stages when the disease has already metastasized. The worst prognosis associated can be attributed to its spread to the vital organs.^3^ Metastasis encompasses a series of well-concerted events where change in cell–cell and cell–extracellular matrix (ECM) interaction has a very crucial role. Complex network of sugar residues (glycocalyx) comprises a major part of the ECM, mediating various social events like cellular adhesion, motility and signaling.^4^ Although recent advancement of glycomics reportedly link aberrant glycosylation with cancer progression^4, 5, 6, 7, 8, 9^ albeit necessary insights emphasizing its importance in the perspective of progression of OC are still not available.

Among the various glycosylation types available in nature, sialylation needs a special mention pertaining to its correlation with metastatic transformation.^10, 11, 12, 13^ Sialic acids (Sias) or *N*-acetyl neuraminic acids comprise a diverse lineage of nine-carbon monosaccharide family executing a wide variety of biological functions.^14, 15^ They are predominantly positioned at the terminal ends of the oligosaccharide side chains through *α*2,3-, *α*2,6- or *α*2,8-linkages.^9^ Sialylated glycans helps to evade apoptosis, gain metastatic advantage and develop chemoresistance. Lectins constitute a well-known ubiquitous group of multivalent proteins and glycoproteins, performing a plethora of biological functions by binding to specific sugar moieties.^16, 17^ They grabbed the attention of the scientific community owing to their role in inducing apoptosis in various cancer cells and suppressing tumor growth *in vivo*.^17, 18, 19^ A major rationale for developing cancer therapeutics resides in targeting apoptosis. Too little apoptosis results in a scenario whereby cells fail to die^20^ and generate malignancy. Although targeting apoptosis provides a feasible option to restore the damage associated with cancer yet not many insights on the application of such strategies in OC is available. Residing at the cross-road where Lectins serve as anticancer agents, hyper-sialylation provides a strategy for cancer cell survival^11^ and the development of non-toxic, antitumor agents lies as an urgent need, we were prompted to investigate the potency of Lectins in enhancing the sensitivity of OC toward apoptosis.

Speculating the immense impact that Sias may impart upon the spread of OC, we selected the lectin *Sambucus nigra* agglutinin (SNA) to study the status of sialylation of the disease. This lectin exhibits specificity toward binding *α*2,6-linked sias.^21, 22^ The strong binding of SNA to the OC cells was imperative of the heightened expression of surface Sias, which led to triggering of the AKT/ERK pathways. We demonstrated the involvement of the aforementioned signaling axes in the de-phosphorylation of dynamin-related protein-1 (Drp-1) and its subsequent translocation to mitochondria resulting in their fission. This phenomenon induces a shift in the cellular bioenergetics, which finally terminates into the activation of the caspase cascade resulting in apoptosis and cellular demise. Our evidences notably come up with a novel strategy whereby molecules of natural origin can be exploited for their role in curbing the lethality associated with OC.

## Results

### Hyper-sialylation associated with ovarian cancer drives the specificity of SNA

**Figure 1:**
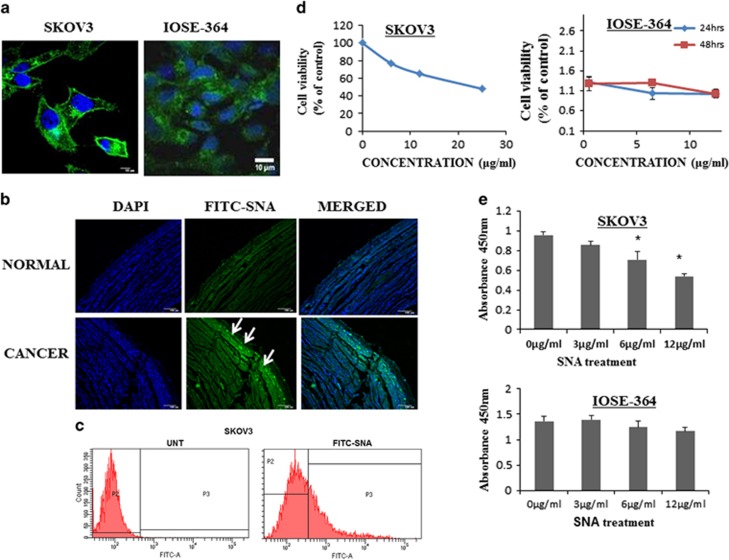
Hyper-sialylation associated with ovarian cancer drives the specificity of SNA. (**a**) Binding of FITC-SNA to the surface of SKOV3 and IOSE-364 was seen through confocal imaging. Scale bar =10 *μ*m (**b**) Immunohistochemical analysis showing binding of FITC-SNA (green, shown by arrow marks) to ovarian tissue sections. The nuclei were stained with DAPI. Bar=100 *μ*m. (**c**) Surface binding of SNA was quantitated by flow cytometry in SKOV3 cells. (**d**) Quantitation of cellular viability was performed using MTT reagent in SKOV3 and WST-1 reagent in IOSE-364 cell lines. (**e**) BrdU proliferation assay was performed in SKOV3 and IOSE-364 cell lines with different doses of SNA as indicated

As OC is associated with enhanced sialylation, we checked the status of Sias on the surface of high-grade serous carcinoma (HGSC) cells SKOV3 and normal ovarian epithelial cells, IOSE-364. Intense fluorescence signal observed along the membrane of SKOV3 as compared with IOSE-364 (Figure 1a) was imperative of the high expression of Sias on the HGSC cells. Non-malignant controls, NIH3T3 and A549 cell lines showed insignificant surface binding (Supplementary Figure 1a). Immunohistochemical studies performed on healthy and OC tissues corroborated with the *in vitro* result (Figure 1b). The binding when quantitated by flow cytometric analysis in SKOV3 (Figure 1c), OAW-42 and IOSE-364 (Supplementary Figure 1b) cells, the former two exhibited enhanced surface binding. To further assess the effect of SNA on cellular viability, SKOV3 cells were treated with serial concentrations of SNA (0, 6, 12 and 25 *μ*g/ml) for 24 h. Minimum dose of 6 *μ*g/ml was sufficient to cause 50% growth inhibition. The comparative dose profile (Figure 1d) of IOSE-364 treated with SNA with indicated doses, showed insignificant effect manifesting the selectivity of SNA. To ascertain the effect of SNA on the cellular proliferation, SKOV3 and IOSE-364 cells were subjected to BrdU proliferation assay in presence of different doses of SNA (Figure 1e). The proliferation rate of IOSE-364 was unperturbed in response to SNA treatment, whereas SKOV3 exhibited a consistent decrease in proliferation. These results together indicated enhanced *α*-2, 6 sialylation occurring in OC cells drive the binding of SNA onto their surface eventually reducing their viability.

### Apoptosis was induced by SNA in OC cells

**Figure 2:**
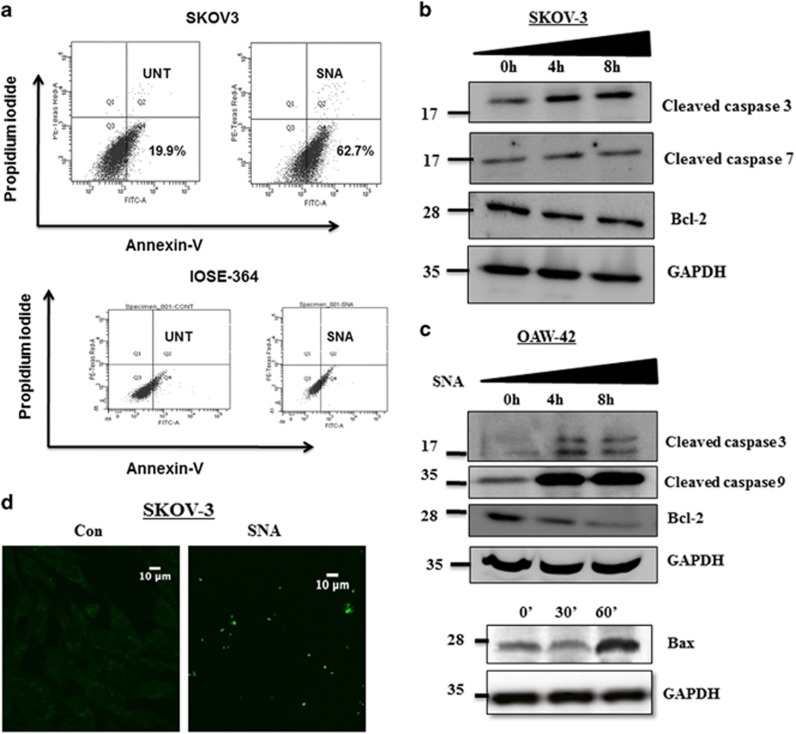
SNA induces apoptosis in OC cells. (**a**) After 24 h of SNA treatment SKOV3 and IOSE-364 cells were analyzed for apoptosis in a FACS flow cytometer. (**b**and**c**) Western blot analysis for cleaved caspase-3, -7, -9, Bax and Bcl-2 was done in SKOV3 and OAW-42 cells using SNA-treated whole cell proteins. GAPDH was used as loading control. (**d**) TUNEL assay was done in SKOV3 cells and then observed by confocal microscopy. Scale bar=10 *μ*m

The seemingly evident interrelation between decreased cellular viability and apoptosis, urged us to investigate the association of SNA with apoptosis. The apoptotic potential of SNA was quantified using Annexin-V and propidium iodide (PI) staining (Figure 2a) in SKOV3 cells. We observed significant increase in the Annexin-V-positive population from 19.9% in untreated to 62.7% after 24 h of SNA treatment. The appearance of apoptotic population in IOSE-364 could be attributed to its basal SNA binding, which was insignificant in comparison with SKOV3. The increased expression of pro-apoptotic Bax and the decrease in anti-apoptotic Bcl-2 after SNA treatment (Figures 2b and c), shifted the ratio of pro- and anti-apoptotic signal toward apoptosis. This was also accompanied by the elevated levels of cleaved caspase-3 and caspase-9 emphasizing the activation of apoptotic pathway in a time-dependent manner in OAW-42 and SKOV3 cells. The appearance of TUNEL-positive cells after SNA treatment was also an indication of the apoptotic nuclei (Figure 2d). Collectively, these data conclude that the decrease in viability of the cancer cells is attributed to the induction of apoptosis by SNA.

### SNA administration led to mitochondrial dysfunction through enhanced ROS generation and cytochrome-c release

**Figure 3:**
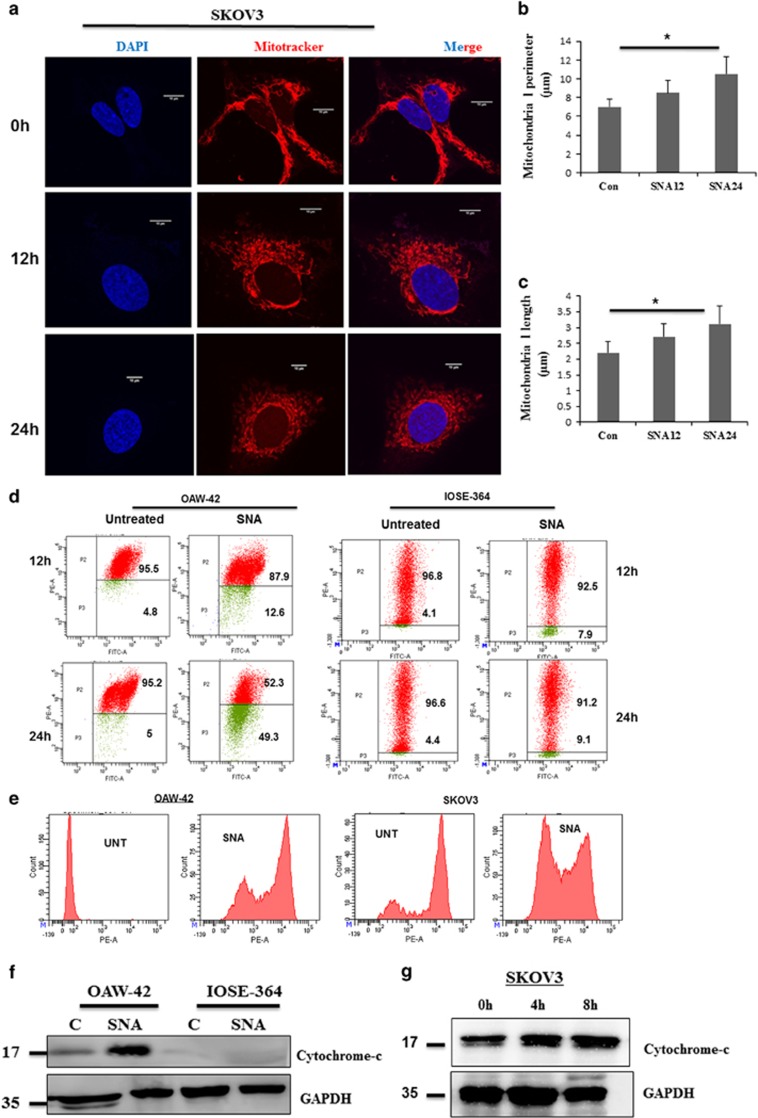
SNA exposure results in mitochondrial dysfunction. (**a**) SKOV3 cells stained with MitoTracker Red CMXROS were imaged for mitochondrial structure by confocal microscope. Scale bar represents 10 *μ*m. (**b-c**) Graphs showing the mitochondrial length and perimeter of SKOV3 cells after indicated time period of SNA exposure. (**d**) Membrane potential of OAW-42 and IOSE-364 cells stained with JC-1 dye were measured by flow cytometry. (**e**) ROS production in OAW-42 and SKOV3 cells stained with Mitosox was analyzed by flow cytometry. (**f**) Western blot depicting cytosolic release of cytochrome-c in OAW-42, IOSE-364 cell lines after 24 h of SNA treatment with GAPDH as loading control. (**g**) Expression of cytochrome-c after 0, 4 and 8 h of SNA treatment in SKOV3 cells with GAPDH as loading control

Induction of apoptosis necessitates several ATP-requiring processes.^23, 24^ As mitochondria are involved in the cellular bioenergetics and regulation of apoptosis,^25, 26, 27^ we focused on their involvement in SNA-induced apoptosis. To visualize the effect of SNA on mitochondrial morphology, we stained SKOV3 (Figure 3a) and OAW-42 (Supplementary Figure 2) cells with mitochondria-specific probe MitoTracker Red CMXROS. Increase in the mitochondrial length and perimeter (Figures 3b and c) was an indication of fragmented mitochondria.

We examined the status of mitochondrial outer membrane potential (MOMP) in SNA-treated OAW-42 and IOSE-364 cells for 12 and 24 h time point by flow cytometry (Figure 3d). A decrease in the MOMP was observed by the switch in the JC-1 fluorescence from red to green in response to SNA. As ROS generation and cytochrome-c production are associated with apoptotic induction,^28, 29^ we checked their status in SNA-treated OAW-42 and SKOV3 cells. SNA enhanced ROS generation (Figure 3e) in both the cell lines. Heightened release of cytochrome-c was observed in the cytosolic fraction of OAW-42 cells but SNA failed to induce similar effects in IOSE-364 cells (Figure 3f). SKOV3 also showed similar increase in cytochrome-c release (Figure 3g). Our findings thus suggested that SNA-induced apoptosis in OC cells was intimately associated with mitochondrial dysfunction.

### SNA induces a shift of cellular respiration toward oxidative phosphorylation

**Figure 4:**
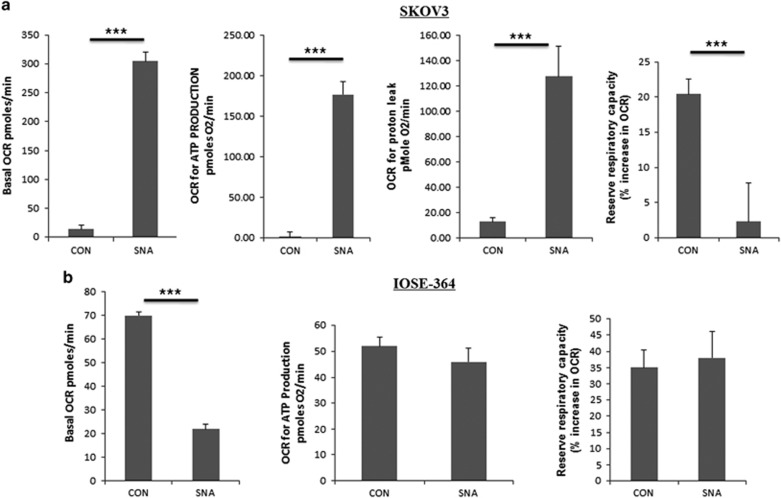
A shift in cellular respiration towards. OXPHOS occurs under SNA exposure. OCR was measured by extracellular flux analyzer (Seahorse Bioscience). The basal OCR ATP production, proton leak and reserve respiratory capacity of untreated and SNA administered SKOV3 (**a**) and IOSE-364 (**b**) cells were measured by XF24 flux analyzer. The data shown here are mean ±S.E.M. for three experiments performed independently

Mitochondria have a pivotal role in determining whether a cell proceeds toward survival or apoptosis.^30^ Metastatic potential is reflected by the dependency of cancer cells on anaerobic glycolysis for energy production often termed as the Warburg effect.^31, 32^ Oxidative phosphorylation (OXPHOS) was quantitated by the oxygen consumption rate (OCR). The bioenergetics of mitochondria in SNA-treated SKOV3 cells were studied by XF-flux analyzer. SNA increased the basal OCR values (Figure 4a), which was indicative of a shift in the cellular respiration toward mitochondrial OXPHOS thereby leading to an increase in the ATP production. An increase in the proton leak, represented loss of mitochondrial membrane integrity. Associated decrease in the reserve respiratory capacity dictates the vulnerability to oxidative stress. IOSE-364 administered with SNA showed a decrease in basal OCR (Figure 4b). The results in OAW-42 (Supplementary Figure 3) were in agreement with SKOV3 data. These results identify the potential of SNA in restoring the cellular bioenergetics in favor of decreasing the metastatic potential.

### Mitochondrial fission promoted by translocation of Drp-1 results in decrease of cellular viability

**Figure 5:**
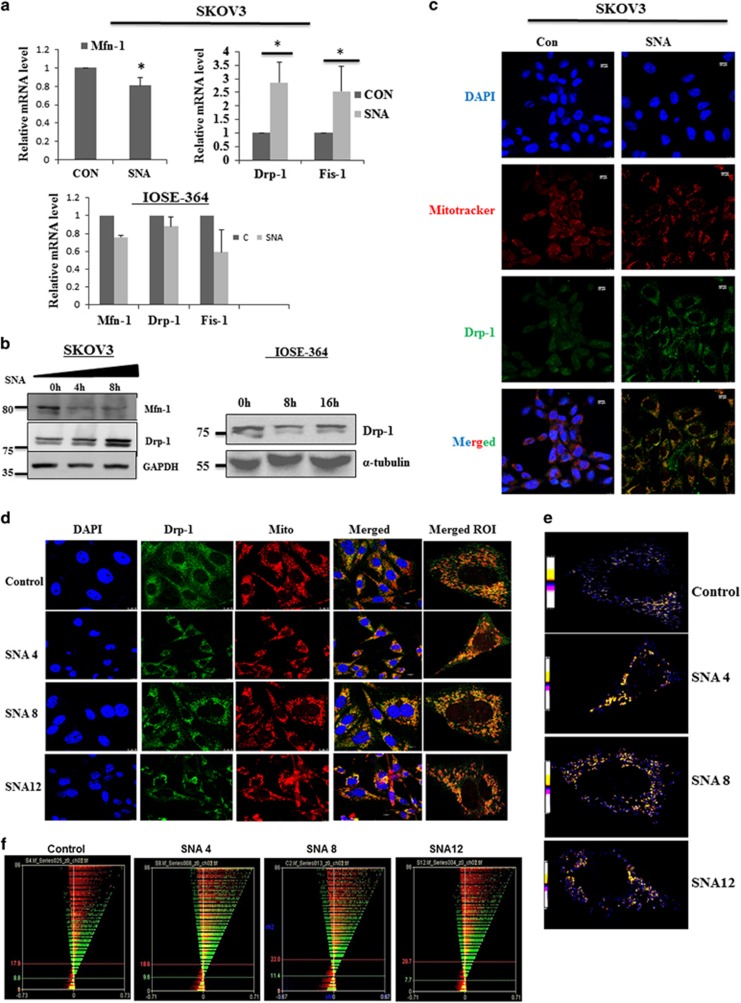

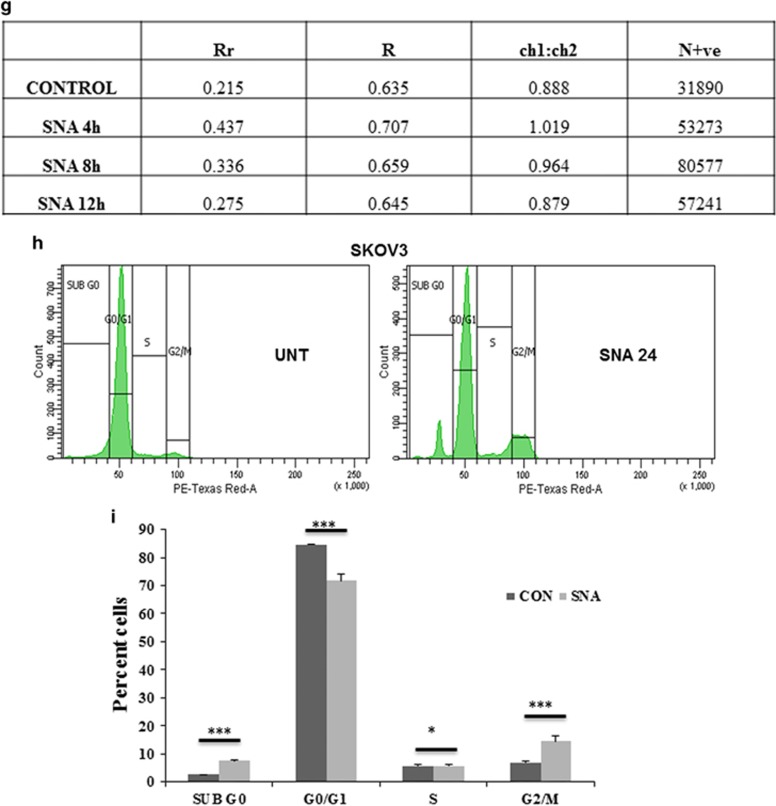
Mitochondrial fission promoted by mitochondrial translocation of Drp-1 upon SNA exposure, results in decrease of cellular viability. (**a**) Q-PCR of mitochondrial fission and fusion genes after 4 h of SNA treatment in SKOV3 and IOSE-364 cells. (**b**) Western blots of Mfn-1 and Drp-1 in SKOV3 cells with GAPDH as loading control and Drp-1 in IOSE-364 cells with tubulin as loading control. (**c**) Confocal microscopy depicting colocalization of Drp-1(green) with mitochondria (red) in SKOV3 after SNA treatment for 8 h. Nuclei were stained with DAPI. Scale bar=10 *μ*m. (**d**) Microscopic images of colocalization of Drp-1 (green) with mitochondria (red) in OAW-42 cells treated with SNA for the mentioned time points. ROI indicates merged region of interest. Scale bar=10 *μ*m. (**e**) Qualitative analysis by PDM imaging. (**f**) ICA plots generated. (**g**) Statistics of colocalization study done by ICA. (**h**) Cell cycle analysis of SKOV3 treated with SNA for longer time periods (24 and 36 h) as observed by flow cytometry. (**i**) Cells quantitated in each phase of cell-cycle represented as bar diagram

Mitochondrion has a crucial function in influencing the dynamicity of cancer cell metabolism. These organelles are in a continuous flux undergoing fusion and fission processes, changing their morphology, which is related to the energy metabolism.^33, 34, 35^ We hypothesized that the shift in bioenergetics may be a downstream effect of SNA-induced morphological changes in mitochondria. The real-time PCR data indicated increase in expression of the fission genes, Drp-1 and mitochondrial fission-1 protein (Fis-1) with a parallel decrease in the fusion gene Mitofusin-1 (Mfn-1) in SKOV3 cells. However, the expression levels merely changed in IOSE-364 (Figure 5a). The results were corroborated in the protein levels, where SNA-induced the expression of Drp-1 in a time-dependent manner with an associated decrease in Mfn-1 in SKOV3 cells. The expression level of Drp-1 remained unchanged in IOSE-364 cells at different time of SNA treatment (Figure 5b).

Mitochondrial fission necessitates the translocation of Drp-1 to mitochondria from cytosol^36, 37^ after being de-phosphorylated at Ser-637. SNA treatment showed mitochondria-specific localization of Drp-1 both in SKOV3 and OAW-42 (Figures 5c and d). The colocalization was confirmed qualitatively by PDM analysis (Figure 5e) and quantitatively by Intensity Correlation Analysis (ICA; Figure 5f). The ICA statistical data (Figure 5g) showed that the increase in the Pearson’s correlation coefficient (Rr) values from 0.215 to 0.437 and 0.336 after 4 and 8 h of SNA treatment, respectively, along with an associated enhancement of overlap coefficient (R) and the number of pairs of pixels having a positive PDM values (N+ve). Altogether these data are a definite indication of the translocation of Drp-1 to mitochondria in response to SNA.

Mitochondrial dynamicity is related to cell cycle progression.^38^ We speculated whether halting the progression through the cell cycle may result in reduction in proliferation. We found that SNA inhibits completion of cell cycle by restricting the cells at G2/M phase (Figure 5h). The cells in each phase of the cell cycle were quantitated and represented as bar diagram (Figure 5i).

### AKT-ERK1/2 pathway has a crucial role in SNA-induced apoptosis in ovarian cancer cells

**Figure 6:**
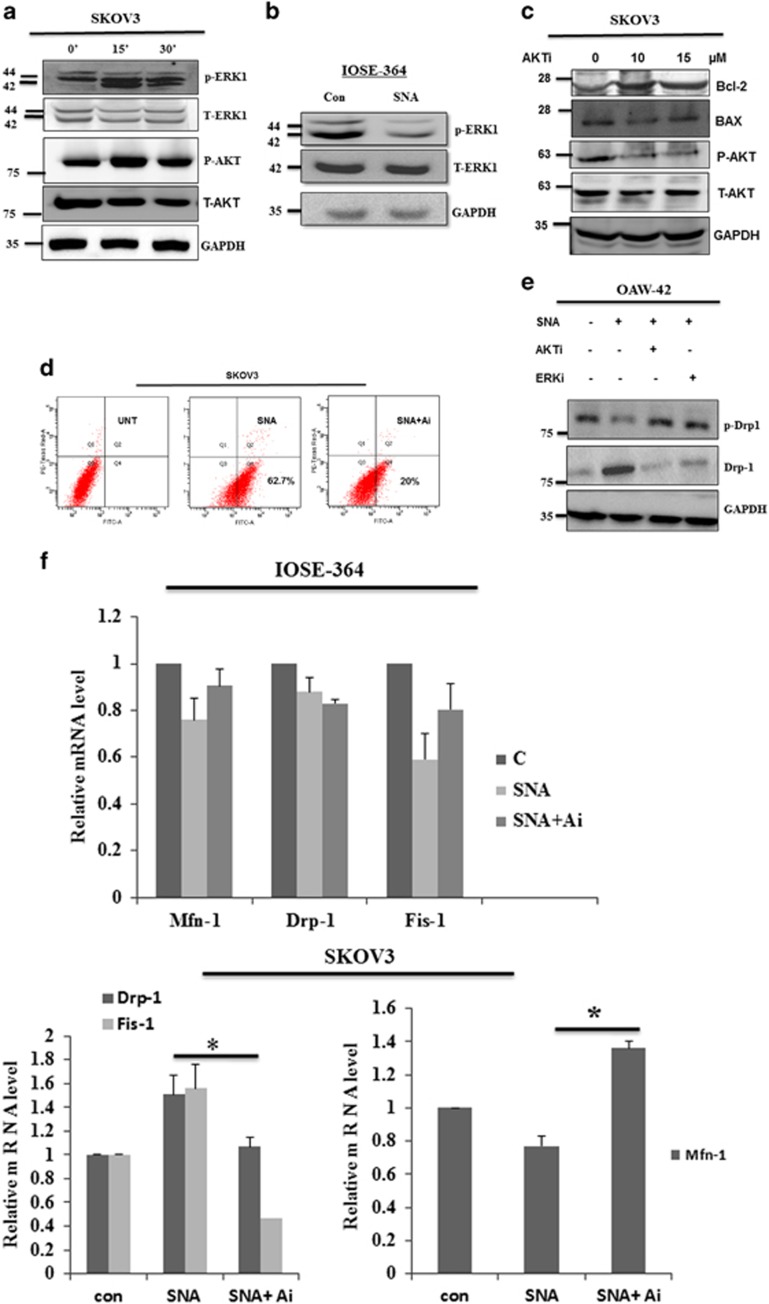
SNA induces apoptosis through activation of the AKT-ERK1/2 pathways. Cells were stimulated with 12 *μ*g/ml of SNA for indicated time periods. (**a** and **c**) Lysates prepared from the SKOV3 cells were analyzed for p-AKT, p-ERK1, T-AKT, T-ERK1, Bax, Bcl-2 with GAPDH as loading control. (**b**) Lysates from IOSE-364 were checked for the expression of p-ERK1 after 30-min incubation and T-ERK1 after 24-h incubation with GAPDH as loading control. (**d**) Apoptotic induction in SKOV3 cells were quantified by flow cytometry after 24 h of SNA treatment in presence or absence of 10 *μ*M AKTi by Flow cytometry. (**e**) OAW-42 cell lysates were analyzed for p-Drp-1 and Drp-1 after 30-min and 24- h incubation with SNA, respectively. GAPDH was used as loading control. (**f**) The Q-PCR of Mfn-1, Drp-1 and Fis-1 after 4 h of SNA treatment was observed in IOSE-364 and SKOV3 cells

To explore the signaling pathways associated, SKOV3 cells were treated with SNA for 0, 15 and 30 min. Enhanced phosphorylation of AKT and p44 in response to SNA suggested the activation of these pathways (Figure 6a). In contrast, IOSE-364 exhibited undetectable amount of p-AKT with marked decrease in p-p44 level after SNA treatment (Figure 6b). Usage of AKT inhibitor (AKTi) alone in SKOV3 cells decreased the expression of pro-apoptotic Bax and increased the expression of anti-apoptotic Bcl-2. This was associated with decreased p-AKT level with unaltered T-AKT expression (Figure 6c). AKTi was capable of inhibiting SNA-mediated apoptosis in SKOV3 cells as the percent apoptotic cells was decreased from 62.7 to 20% upon AKTi treatment (Figure 6d). To validate the relevance of these pathways as it pertains to mitochondrial dysfunction, we checked the status of p-Drp-1 post treatment of cells with pathway inhibitors. As expected, AKTi and ERKi abrogated the de-phosphorylation of Drp-1 (Figure 6e). AKTi was able to impede the SNA-mediated effects on the transcriptional regulation of the mitochondrial fission and fusion genes, namely Drp-1, Fis-1 and Mfn-1, as was evident through the real-time PCR data (Figure 6f). All these results together indicate the involvement of AKT and p44 pathways in SNA-induced apoptosis mediated by mitochondrial dysfunction.

## Discussion

Cancer is a process of 'microevolution'^39^ where the fittest cell gains the survival advantage among a heterogeneous population. Hence it is logical to speculate that carcinomatous cells, which modulate their repertoire of defense mechanisms are bestowed with the advantage of perpetual proliferation and survival. Altered glycosylation and evasion of cell death mechanisms are two such features^40, 41^ by virtue of which cancer cells gain tissue mass and resistance to clinical regimens. Exploitation of these defense strategies for therapeutic gain was the primary goal of our study. Our hypothesis was to translate the mechanistic details underlying the disease to identify an innocuous therapeutic lead to help improving clinical interventions. Differential surface expression of glycans helps cancer cells in gaining invasive advantage.^42, 43^ Evidences suggest, increase in the endogenous levels of sialylated glycoproteins especially *α*2,6-linked Sias, correlate with poor prognosis and survival rate of carcinoma patients.^44, 45^ This unique profile of glycan epitopes can thus be used as ideal candidates to be targeted by specific bait proteins, lectins. In spite of the association of OC with a number of altered glycosylation fates^46, 47^ including sialylation, not many mechanistic insights are available.

Conventional therapeutics lacking specificity, impart severe damage to the normal cells restricting their clinical efficacy. Agents harboring the capacity to specifically target cancer cells, have the potential to be developed as a potent antitumor drug. To the first of our knowledge, we have shown that SNA exhibits selective recognition of cancer cells (Figure 1). Tumor cells resist the available therapeutic challenges by evading apoptosis. Targeting the cells to induce apoptosis may be a key strategy to combat cancer progression. Lectins are known to induce apoptosis in a variety of cancer cells.^48, 49, 50^ That SNA was successful in doing so was corroborated with our findings (Figure 2).

Mitochondria being the hub of energetic functions have the cue to the life and death of a cell.^30, 51^ Cell survival rests on various critical functions of the mitochondrial membrane, which undergoes considerable morphological changes in the initial stages of apoptosis.^52, 53^ To understand the effect of SNA on OC cells, we noted the active involvement of mitochondria. A decrease in MOMP and enhanced ROS generation upon SNA treatment (Figure 3) was an indication of mitochondrial dysfunction normally seen in activation of the intrinsic apoptotic pathway.^53^ Mitochondria switch their shapes in response to the metabolic demands of the cell.^54^ Reduction in the energy supply leads to the mitochondrial fragmentation releasing cytochrome-c into the cytosol, which then activates the caspase cascade leading to cell death.^30^ The activation of caspases along with the appearance of enhanced cytochrome-c in the cytosolic fraction indicated SNA-induced mitochondrial membrane permeabilization (Figure 3). Fission per se does not necessarily indicate apoptosis although it has a fundamental role in cell death. The translocation of Drp-1 from cytosol to mitochondrial fission loci remains the crucial mediator of fission, as was evident by our fluorescence imaging studies (Figure 5).

Metabolic alterations remain the key step that helps in the transformation of a normal cell into a tumor phenotype.^55^ Cancer cells rely mostly on anaerobic glycolysis for energy supply.^31, 56^ To restrict the excess energy flow needed to support growth of tumor cells, a shift of cellular respiration from glycolysis to OXPHOS stands inevitable. Application of agents that inhibit or perturb the bio-energetic profile of cancer cells would be helpful in the treatment of the disease. Our results indicate SNA was successful in inducing this shift leading to increased basal OCR and ATP production (Figure 4). This confirms the efficiency of SNA in decreasing the metastatic potential of OC cells. Prolonged inadequacy to meet the energy demands of the cell leads to cell cycle arrest. We sought to determine whether the perturbed cellular bioenergetics imparted similar effects or not. The fall in the reserve respiratory capacity (Figure 4) purely indicates the inability of cellular energy pool to meet the sudden burst of energy crisis experienced by the cellular environment, which may also drive the cells to senescence or death. SNA restricts the completion of the cell cycle by arresting the cells before the G2/M phase (Figure 5).

Various signaling pathways have a decisive role in modulating the course of cell survival.^57^ But unlike mitogen-mediated activation, these pathways are also known to be involved in apoptosis induction under the stimulus of specific lectins.^58^ Our observations were in agreement, showing phosphorylation of AKT/ERK pathways in response to SNA (Figure 6). Abrogation of Drp-1 de-phosphorylation on application of the pathway inhibitor suggested active involvement of these signaling axes in SNA-induced cell death (Figure 6).

**Figure 7:**
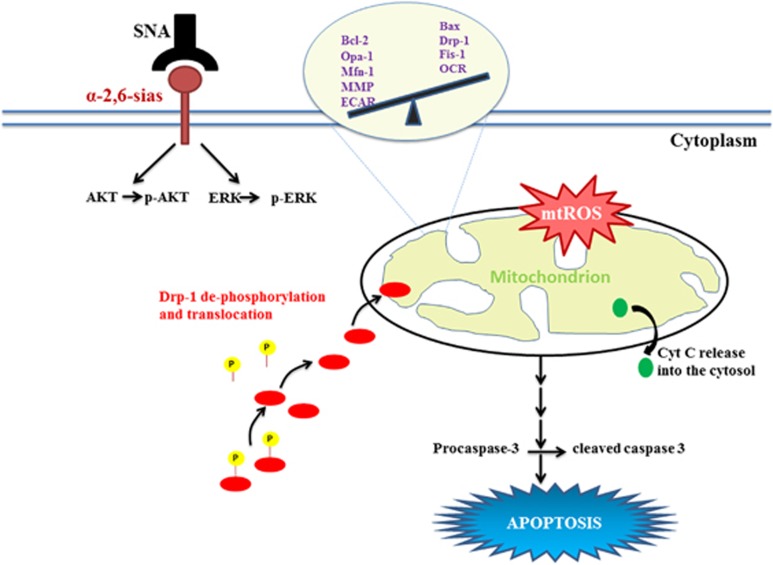
Schematic representation of SNA-mediated induction of apoptosis in OAW-42 cells. Hyper-sialylation associated with OC drives SNA binding to these cells leading to the activation of AKT and ERK1/2 pathways. Meanwhile in response to SNA administration, mitochondrial membrane permeabilization occurs in association with cytochrome-c release into the cytosol and ROS generation leading to mitochondrial dysfunction. The resulting shift in the cellular bioenergetics promotes cell cycle arrest finally culminating into apoptosis via caspase activation

The primary rationale for developing cancer therapeutics lies in designing agents selectively targeting cancer cells. Our objective was to exploit strategies evolved by tumor cells to escape cellular demise. The key finding here remains the identification of the selectivity exhibited by SNA toward binding cancer cells and promoting apoptosis (Figure 7). Implementation of SNA for targeted delivery to the cancer cells can be exceedingly promising in reducing the heavy damage incurred by the normal cells during conventional clinical interventions. Notably our findings encourage identification of SNA-like natural molecules to exploit their efficacy as antitumor drugs resulting in minimal toxicity to the normal cells. This study strongly reckons the relatively nascent arena of targeting mitochondria as an elegant therapeutic approach to reduce the mortality rate of OC patients.

## Materials and methods

### Cell culture

The epithelial ovarian adenocarcinoma cell line (OAW-42), p53 null OC cell line (SKOV3), normal epithelial ovarian cell line (IOSE-364), mouse fibroblast cell line (NIH3T3) and lung carcinoma cell line (A549) were used in this study. OAW-42 and SKOV3 were purchased from Sigma Aldrich, St. Louis, MO, USA and maintained in DMEM (Invitrogen, Carlsbad, CA, USA) and RPMI (Gibco, Waltham, MA, USA), respectively, supplemented with 10% heat inactivated fetal bovine serum (FBS; Invitrogen), 100 mg/ml streptomycin and 100 u/ml penicillin (Invitrogen), at 37 °C in 5% CO_2_ and 95% humidified air. Human immortalized ovarian surface epithelial cells IOSE-364 (a kind gift from Dr. N Aueresperg and Clara Salamanca, Vancouver, Canada) was maintained in MCDB-105 (Sigma Aldrich, St. Louis, MO, USA) and Medium-199 (Invitrogen) in 1 : 1 ratio and supplemented as stated earlier. NIH3T3 (a kind gift from Dr. Debabrata Biswas, IICB, Kolkata, India) and A549 (a kind gift from Dr. Arun Bandopadhyay, IICB True Campus, Kolkata, India) were maintained in DMEM (Invitrogen) with supplementation as mentioned earlier.

SNA (Sigma Aldrich) was directly added into the cells in incomplete medium, initially at 6, 12 and 25 *μ*g/ml. Finally, the optimized concentration of 12 *μ*g/ml was used for time periods as mentioned in the figure legends. The AKT and ERK1/2 inhibitor were purchased from Calbiochem (San Diego, CA, USA) and used at 10 *μ*M final concentration. Before each treatment, cells were transferred to incomplete medium containing 0.5% FBS.

### Confocal microscopy

Immunofluorescence microscopy was done by staining cells grown on cover-slips with FITC-conjugated SNA (12 *μ*g/ml) for 1 h at room temperature followed by standard protocol as depicted previously.^59^ Unbound lectin was washed away with PBS and cells were then stained with 4, 6-diamidino-2-phenylindole DAPI (1 mg/ml) for 5 min before mounting, using mounting medium. Stained cells were visualized by Andor spinning disk confocal microscope (Andor Technology PLC, Belfast, Ireland) using Andor iXON3 ultra EMCCD camera. For mitochondrial fission analysis, cells were stained with MitoTracker Red CMXRos (50 nM, Molecular Probes, Waltham, MA, USA) for 30 mins at 37°C to stain the mitochondria. Immunostaining was done using Drp-1 antibody (1:50, Santa Cruz Biotechnology, Dallas, TX, USA) followed by Alexa Fluor 488 conjugated secondary antibody as mentioned earlier^59^ and images were acquired by Leica STED confocal microscope (TCS SP8, Buffalo Grove, IL, USA) with 60 × magnification.

### Image analysis

ImageJ 'Mitophagy' pluggin and WCIF ImageJ software (Wright Cell Imaging Facility) were used to study mitochondrial morphology and the mitochondrial localization of drp-1. Before analysis, images were converted to 16-bit grayscales. The lengths of the mitochondria were measured by the 'Mitophagy' application. For each time frame, approximately 100 mitochondria from at least 10 different cells were analyzed for the different morphological parameters. ICA method was used to compare the subcellular localization of Drp-1 upon different time intervals of SNA treatment. It depicts the synchrony of intensity between the two channels used here – red and green. Image of (PDM): the product of the differences from the mean, that is, for each pixel: (red intensity─red mean intensity) × (green intensity─green mean intensity) defined colocalization qualitatively. The orange pixels showed colocalization of Drp-1 with mitochondria and blue color illustrates segregation. On the ICA plots, X axis indicated PDM values and y axis denotes red or green intensity. A merged ICA plot of the individual red and green ICA plots quantitatively demonstrates the levels of colocalization. Pearson’s correlation coefficient (Rr), overlap coefficient (R), red:green pixel ratio (Ch1:Ch2) and the number of pixel pairs that have a positive PDM value (N+ve), were generated by the intensive correlation analysis program (http://www.uhnresearch.ca/ facilities/wcif/imagej).

### Immunohistochemistry

Tissue samples were provided by Department of Pathology, Institute of Post Graduate Medical Education and Research, Kolkata, India. Tissues were sectioned as mentioned earlier.^60^ Sectioned tissues were blocked in 3% BSA in 1X PBS-T for 1.5 h and incubated overnight with FITC-SNA (12 *μ*g/ml) at 4 °C. The slides were washed with 1X PBS-T in succession with DAPI staining (1 mg/ml) for 5 min finally followed by mounting on to glass slides. Images were captured using Andor spinning disk confocal microscope (Andor Technology PLC, Belfast, Ireland).

### Fluorescence-assisted cell sorting

Treated cells were fixed with 4% formaldehyde for 10 min at 37 °C and kept on ice for 5 min. In all, 1 × 10^6^ cells were washed, re-suspended and blocked in incubation buffer (0.5 gm BSA in 100 ml PBS) for 10 min. FITC-SNA (12 *μ*g/ml) was used to incubate the cells for 1 h at room temperature and the binding of SNA was quantitated by LSRFortessa cell analyzer (Becton-Dickinson, San Jose, CA, USA).

FITC-Annexin-V staining was used to determine lectin-induced phosphatidylserine externalization of ovarian cancer cells by 'Apoptosis kit' (Molecular Probes) as per manufacturer’s protocol. Percentages of cells positive for Annexin-V and PI individually or in combination were detected by flow cytometry and the dot blot analysis was done using Cell Quest Pro software (BD Biosciences, San Jose, CA, USA). Cells were treated in presence or absence of SNA for 12 and 24 h. MOMP was estimated by staining the cells with the fluorescent dye JC-1 (5*μ*g/ml,) at 37 °C for 15 min. Cells were washed with PBS and analyzed on a flow cytometer for red and green fluorescence.

### Growth inhibitory studies

In all, 5 × 10^4^ cells were seeded in 96-well plates and grown in complete medium allowing 80% confluency. Cells were transferred in incomplete medium with 0.5% FBS and treated with SNA at different concentrations (6, 12 and 25 *μ*g/ml) for different time points (24 and 48 h), maintained in humidified atmosphere (37 °C, 5% CO_2_). After each time point, 10 *μ*l of MTT (5 mg/ml) was added to each well followed by dissolution in 100 *μ*l of DMSO. Finally, absorbance was measured at 550 nm using ELISA plate readers (Winooski, VT, USA). Cell proliferation reagent WST-1 (Roche Diagnostics, Indianapolis, IN, USA) was also used for viable cell detection by following the manufacturer’s protocol and measuring absorbance at 440 nm. Percentages of viable cell were calculated, with respect to controls considered as 100%.

### BrdU proliferation assay

In total, 1 × 10^5^cells/ml were plated onto 96-well culture dish. Cells were then treated with 0, 3, 6 and 12 *μ*g/ml of SNA for 24 h. The assay was performed using BrdU proliferation kit (Sigma, St. Louis, MO, USA) following the manufacturer’s protocol. The final absorbance was measured at 450 nm in a plate reader.

### Western blotting

Cells were treated with SNA for different time intervals, lysed using RIPA lysis buffer. Western blot was performed as previously described.^60^ Bax, Bcl-2, Mfn-1, p-Drp-1(serine 637), Drp-1, GAPDH, p-ERK1 and ERK antibodies were purchased from Santa Cruz Biotechnologies (Dallas, TX, USA) and used at dilution 1:1000. Cleaved caspase-3, cleaved caspase-7, cleaved caspase-9, AKT and p-AKT antibodies were from Cell Signaling Technologies (Danvers, MA, USA) used at dilution 1:2000. Treated cells were extracted for their nuclear and cytoplasmic protein fractions using ProteoJET cytoplasmic and nuclear protein extraction kit (Fermentas, Cleveland, OH, USA) and then blotted using Cytochrome-c and GAPDH antibodies from Santa Cruz biotechnologies.

### TUNEL assay

Seeded cells were allowed to reach 70% confluency and then treated with vehicle control and SNA (12 *μ*g/ml) for 24 h. The TUNEL reaction was carried out using '*In situ* cell death detection kit, fluorescein' (Roche Diagnostics, Mumbai, India) according to the manufacturer's instruction. The images were taken by the confocal microscopy as mentioned above.

### Mitochondrial ROS generation

Flow cytometric analysis of mitochondrial ROS generation was performed by staining control and SNA (12 *μ*g/ml) treated cells with the intra-vital dye Mitosox using gating criteria based on forward scatter, an indicator of size by LSRFortessa cell analyzer (Becton-Dickinson, San Jose, CA, USA). Cells were incubated with the MitoTracker Red CMXROS (Molecular Probes, Waltham, MA, USA, concentration 300 nM) for 40 min at room temperature.

### Measurement of mitochondrial respiration by XF-flux analyzer

Cells were counted with TC-10 cell counter (Bio-Rad, Hercules, CA, USA) and plated at 20 000 cells per well density on XF24 plates. Cells were grown for 24 h in a CO_2_ incubator at 37 °C. One hour before the measurements on an XF24 extracellular flux analyzer (Seahorse Bioscience, Billerica, MA, USA), cells were removed from the CO_2_ incubator and placed at 37 °C in a non-CO_2_ incubator, and media was replaced with 500 *μ*l XF assay media composed of 143 mM NaCl, 5.4 mM KCl, 0.8 mM MgSO_4_, 0.91 mM Na_2_HPO_4_, 2 mM glutamine, 2 mg/ml BSA and 15 mg/l phenol red, pH 7.4. Stock solutions (× 10) of oligomycin, FCCP and rotenone were prepared in XF assay media and loaded into injection ports A, B and C, respectively. Measurements were obtained at 37 °C. The calculations were done as follows: basal OCR=(measurement before oligomycin addition)–non-mitochondrial; proton leak=(first measurement after oligomycin injection through measurement before FCCP)–non-mitochondrial; ATP production=basal respiration–proton leak; and reserve respiratory capacity=maximal respiration–basal respiration.

### Quantitative real-time PCR

Total RNA was isolated from cell lines using TRI-reagent (Sigma) following the standard protocol succeeded by cDNA synthesis from 1 *μ*g RNA using iScript (Fermentas, Cleveland, OH, USA). Q-PCR was performed with fluorescent Power SYBR Green-I on the ABI 7500 Real-Time PCR system (Applied Biosystems, Foster City, CA). 18s levels were used as loading control. The primers used were as follows: human 18s forward –5′-GATTCCGTGGGTGGTGGTGC-3′ and reverse 5′-AAGAAGTTGGGGGACGCCGA-3′, human Drp-1 forward – 5′-AGCGGCAAATCAAACGTCTAG-3′ and reverse –5′-TTGCATTTCCTCA-TGAACCAGTT-3′, human Fis-1 forward –5′-TACGTCCGCGGGTTGCT-3′ and reverse – 5′-CCAGTTCCTTGGCCTGGTT-3′ and human Mfn-1 forward – 5′-GCAACTGAAAAACTGAGGATGATTG-3′ and reverse – 5′-CACAGGCGAGCAAAAGTGGTA-3′.

### Cell cycle analysis

Cells were seeded in six-well plates at a density of 2 × 10^6^ cells per well and treated with SNA for 24 h. Adherent cells were trypsinized and washed, followed by fixation in 70% ethanol overnight at −20 °C. After centrifugation, pellets were re-suspended in 500 *μ*l 1X PBS containing PI (Sigma) working stock (50 *μ*g/ml PI, 0.1 mg/ml RNase A added to PBS) and incubated for 10–15 min before being analyzed by FACS (BD Biosciences, San Jose, CA, USA).

### Statistical analysis

Statistical analysis was performed using Microsoft excel. Data are shown as mean±S.D. of at least three independent experiments. Significant difference between groups with equal numbers was analyzed by two-sided Student's *t*-test. Correlation between groups of variables was analyzed with Pearson’s correlation. *P*-values <0.05 were considered statistically significant. *P<0.05, **P<0.05, and ***P<0.0005.
