# Supplementary material for: Targeted apoptosis in ovarian cancer cells through mitochondrial dysfunction in response to Sambucus nigra agglutinin
Source: Cell Death Dis. 2017 May 4;8(5):e2762–. doi: 10.1038/cddis.2017.77 (PMC5520748; doi:10.1038/cddis.2017.77)
Supplement: Supplementary Information [file cddis201777x1.pdf]

Supplementary Information:

Targeted apoptosis in ovarian cancer cells through mitochondrial dysfunction in response to *Sambucus nigra* agglutinin

Running title: SNA mediates apoptosis through mitochondrial dysfunction

Shreya Roy Chowdhury[1], Upasana Ray[1], B. P. Chatterjee[2] and Sib Sankar Roy[1]*

[1]Cell Biology and Physiology Division, CSIR-Indian Institute of Chemical Biology, Council of Scientific and Industrial Research, 4 Raja S. C. Mullick Road, Kolkata 700032, India.
[2]Department of Natural Sciences, West Bengal University of Technology, Salt Lake, Kolkata, 700064, India.

*Correspondence:

Mailing address: Cell Biology and Physiology Division,
CSIR-Indian Institute of Chemical Biology,
4 Raja S. C. Mullick Road,
Kolkata 700032, India.

Tel : + 91-33-2499-5858

Fax : + 91-33-2473-5197

Email: sibsankar@iicb.res.in

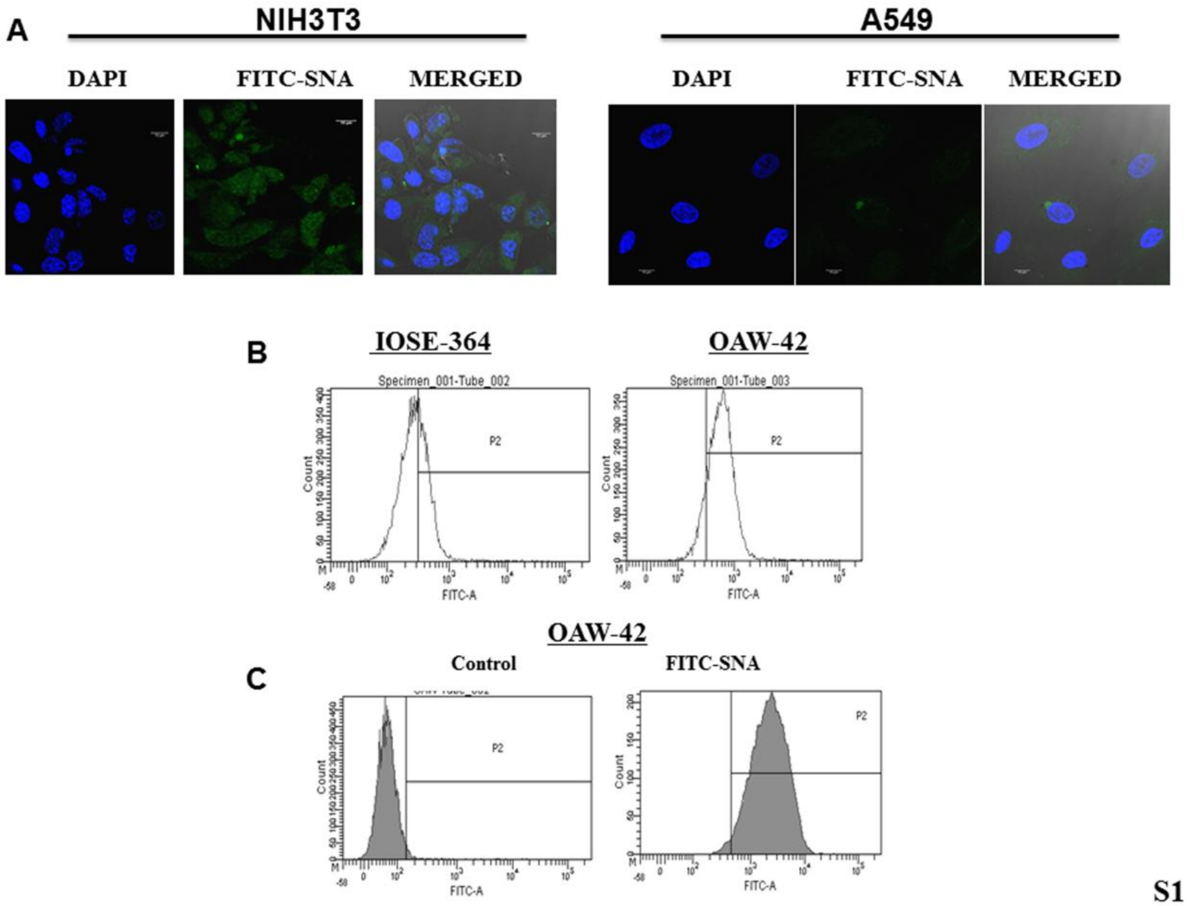

S1

Supplementary Figure Legends:

S1: OC cells are associated with enhanced sialylation. (A) Confocal microscopy of NIH3T3 and A549 cells stained with DAPI and FITC-SNA. Scale bar =10µm. (B) Quantitation of FITC-SNA binding by flow cytometry in IOSE-364 and OAW-42 cells. (C) Surface binding of SNA was quantitated by Flow-cytometry in OAW42 cell lines.

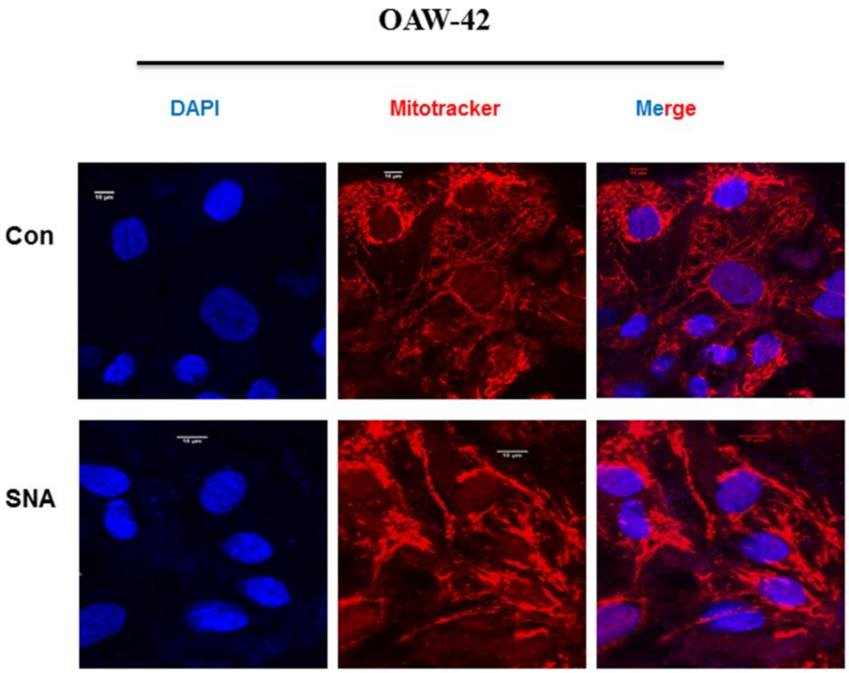

S2

S2: SNA treatment led to fragmented mitochondrial morphology in OAW-42 cells. OAW-42 cells were stained with MitoTracker Red CMXROS and imaged for mitochondrial morphology by confocal imaging. Scale bar represents 10 μm.

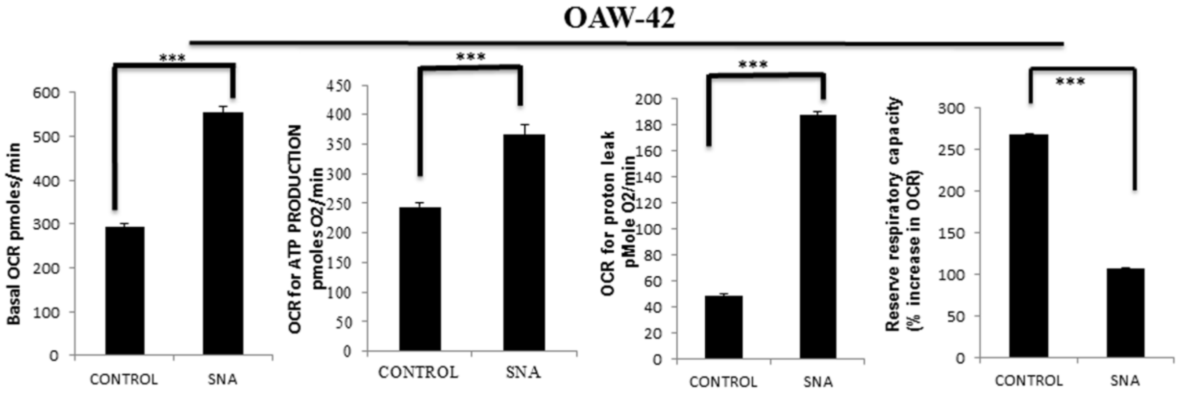

S3

S3: SNA-treated OC cells exhibited shift in cellular bioenergetics. Oxygen Consumption Rate (OCR) was measured in SNA-treated OAW-42 cells by extracellular flux analyzer (Seahorse Bioscience). The data shown here are mean ±SEM for three experiments performed independently.
